# EEG-Based Emotion Estimation Model Integrating Structural and Time-Series Information Based on Deep Learning Architecture Optimization

**DOI:** 10.3390/s26041210

**Published:** 2026-02-12

**Authors:** Kota Tsuji, Keiko Ono, Takuya Futagami

**Affiliations:** 1 Graduate School of Science and Engineering Information and Computer Science, Doshisha University, Kyoto 610-0394, Japan; tsuji.kota@mikilab.doshisha.ac.jp; 2 Department of Intelligent Information Engineering and Sciences, Doshisha University, Kyoto 610-0394, Japan; tfutagam@mail.doshisha.ac.jp

**Keywords:** emotion estimation, EEG, GCN, LSTM, DARTS, DEAP, four-class classification

## Abstract

Emotion recognition is increasingly important for applications in mental health and personalized marketing. Traditional methods based on facial and vocal cues lack robustness due to voluntary control, motivating the use of EEG signals that capture neural dynamics with high temporal resolution. Existing EEG-based approaches using CNNs and LSTMs have improved spatial and temporal feature extraction; however, they still face critical limitations. These models struggle to represent electrode connectivity and adapt to inter-individual variability, and their architectures are typically handcrafted, requiring extensive manual tuning of hyperparameters and structural design. Such constraints hinder scalability and personalization, highlighting the need for automated architecture optimization. To address these challenges, we propose a dual-pipeline architecture that integrates frequency-domain and time-domain EEG features. The frequency-domain branch employs a Graph Convolutional Network (GCN) to model spatial relationships among electrodes, while the time-domain branch uses LSTM enhanced with Channel Attention to emphasize subject-specific informative channels. Furthermore, we introduce Differentiable Architecture Search (DARTS) to automatically discover optimal architectures tailored to individual EEG patterns, significantly reducing search cost compared to manual tuning. Experimental results demonstrate that our framework achieves competitive accuracy and high adaptability compared to state-of-the-art baselines, marking the first integration of GCN, LSTM, channel attention, and architecture search for EEG-based emotion recognition.

## 1. Introduction

Emotion recognition has gained significant traction across multiple disciplines, extending from psychiatric diagnostics to the optimization of consumer engagement in marketing [1]. Traditional methods primarily rely on facial expressions and vocal cues to infer affective states. These non-invasive approaches offer significant practical advantages, as they require no specialized equipment such as neurocaps or facial attachments, making them highly suitable for measurements in naturalistic environments and everyday settings [2]. However, these modalities present fundamental limitations: They capture only overt behavioral manifestations that individuals can intentionally alter or mask, and they are insufficient for accessing genuine internal emotional states that may not be reflected in observable expressions or vocal patterns. Consequently, the robustness and ecological validity of such systems are compromised, particularly in applications requiring genuine emotion detection. In response to these limitations, there has been growing interest in incorporating deeper physiological signals—such as electroencephalography (EEG), electrocardiography (ECG), electrodermal activity (EDA), and electromyography (EMG)—which reflect autonomic nervous system activity, are less amenable to voluntary manipulation, and provide more direct access to genuine internal emotional states [3]. Among these modalities, EEG has attracted considerable attention because it directly captures brain activity at high temporal resolution, enabling analysis of the neural dynamics underlying emotional states. This capability makes EEG-based approaches particularly promising for developing objective and reliable emotion recognition systems.

Various studies have been conducted in the past focusing on EEG-based emotion recognition. For instance, numerous approaches have employed Convolutional Neural Networks (CNNs) to extract discriminative features from EEG signals [4,5,6,7]. CNNs are particularly well-suited to this task because they can automatically learn hierarchical spatial patterns from raw or minimally processed EEG data, reducing the need for handcrafted features. Moreover, CNNs effectively capture local dependencies and spatial correlations across electrode channels, which are critical for modeling the complex brain dynamics underlying emotional states. This capability has led to significant improvements in classification accuracy compared to traditional machine learning methods.

However, CNNs inherently distort spatial relationships when projecting 3D electrodes onto 2D grids. While Graph Convolutional Networks (GCNs) resolve this by directly modeling topological connections, they remain primarily spatial models and—like CNNs—struggle to capture the temporal dependencies inherent in EEG signals. To address this limitation, Long Short-Term Memory (LSTM) networks have been introduced, offering a mechanism to model sequential dynamics by maintaining long-range temporal context through gated recurrent units [8,9]. LSTM models are particularly advantageous for EEG-based emotion recognition because emotional states often manifest as temporal patterns rather than isolated spatial features. By leveraging memory cells and gating mechanisms, LSTMs can effectively learn these temporal dependencies, leading to improved performance in scenarios where the evolution of brain activity over time is critical.

Recent advances in deep learning have introduced attention-based architectures, such as transformers, which leverage self-attention mechanisms to capture long-range temporal dependencies more effectively than traditional recurrent networks. While these models have shown promise in various sequential tasks, Walther et al. [10] highlight a practical challenge in EEG-based emotion recognition: achieving optimal performance with transformers typically requires substantial amounts of training data, which is often limited in clinical settings. Moreover, standard transformer architectures tend to learn electrode relationships as data-driven attention weights without explicitly encoding the topological constraints of brain networks or the 3D spatial configuration of the scalp.

To jointly address spatial and temporal characteristics of EEG signals under such constraints, sequential GCN-LSTM frameworks have been explored [11,12]. These approaches employ graph convolutions to explicitly model spatial relationships among electrodes, followed by recurrent networks to capture temporal dynamics. However, the serial nature of this processing introduces an information bottleneck: the initial GCN stage compresses spatial features into abstract representations, potentially discarding fine-grained temporal variations critical for subsequent LSTM processing. Furthermore, frequency-domain features commonly used with GCNs may attenuate transient temporal dynamics that LSTMs excel at capturing.

Beyond architectural considerations, prior research has explored strategies to enhance clinical feasibility by considering the differential importance of EEG electrodes [13]. Approaches such as focusing on symmetric regions or selecting electrodes most strongly associated with emotional processing have been proposed to reduce complexity and improve practicality in real-world applications. However, these methods face significant challenges due to substantial inter-individual variability in brain activity patterns. This variability makes it difficult to generalize electrode selection across subjects, and incorporating such personalized configurations into deep learning models remains an open research problem [3,14].

We hypothesize that these challenges can be mitigated by a model that automatically learns both temporal dynamics and spatial relationships among electrodes while adaptively emphasizing subject-specific important channels. To achieve this, as a first novelty, we propose a dual-pipeline architecture that simultaneously processes frequency- and time-domain features to fully exploit the rich information in EEG signals. In the frequency-domain branch, a Graph Convolutional Network (GCN) [15] is employed to model the spatial relationships between electrodes by leveraging their connectivity patterns, which are represented as a graph structure. This enables the network to aggregate information from neighboring electrodes and capture structural dependencies critical for emotion recognition. In parallel, the time-domain branch uses an LSTM, which excels at modeling sequential dependencies, to capture the temporal dynamics of emotional states. Furthermore, as a second novelty, to account for subject-specific variability and improve robustness, we incorporate electrode importance scores computed by a Channel Attention (CA) mechanism into the LSTM input. This attention-based weighting emphasizes features from the most informative electrodes, allowing the model to adaptively focus on regions that contribute most to emotion recognition.

Moreover, as a third novelty, we address the challenge of hyperparameter optimization. Maximizing emotion recognition accuracy for each individual requires an efficient mechanism to automatically identify an optimal architecture tailored to subject-specific characteristics. Given the substantial variability in EEG patterns, hyperparameter optimization is essential; however, manual search is computationally expensive and impractical. To overcome this, we adopt Differentiable Architecture Search (DARTS), which formulates architecture selection as a differentiable optimization problem. By defining candidate operations with varying structural hyperparameters and jointly optimizing architecture parameters and model weights, DARTS enables automatic discovery of subject-specific architectures at significantly lower cost compared to conventional evolutionary or reinforcement learning-based methods [16]. To the best of our knowledge, no prior work has incorporated DARTS into EEG-based emotion recognition frameworks, making this study the first to explore this direction. The main contributions are summarized as follows:

1. **Dual-Pipeline Graph-Based Architecture**: We propose a framework that processes frequency-domain and time-domain EEG features in parallel, using GCN to capture spatial connectivity and LSTM to model temporal dynamics.
2. **Channel Attention for Personalization**: We introduce a Channel Attention mechanism to automatically identify and emphasize subject-specific important electrodes, improving robustness and adaptability.
3. **Automated Architecture Optimization with DARTS**: We employ Differentiable Architecture Search (DARTS) to automatically discover subject-specific optimal architectures, reducing search cost significantly.

## 2. Related Work

Research on EEG-based emotion recognition has evolved significantly over the past decade [1,17]. Existing studies can be broadly categorized into three main directions: (1) deep learning architectures for spatiotemporal feature learning, (2) electrode selection and personalization strategies, and (3) automated architecture optimization.

Early approaches relied on traditional machine learning classifiers, which often struggled to model complex nonlinear patterns. In contrast, recent work has shifted toward Deep Learning architectures that can extract high-level, hierarchical features. Convolutional Neural Networks (CNNs) have been widely adopted for their ability to capture spatial correlations across EEG channels. For instance, Yanagimoto et al. [4] proposed a CNN-based model for emotion recognition, demonstrating improved accuracy over traditional classifiers. Similarly, Phan et al. [5] introduced a multi-scale CNN architecture to extract features at different resolutions, enhancing robustness against noise. Building on this direction, Li et al. [18] recently proposed an enhanced DenseNet model integrating multi-scale convolutional kernels to improve the reuse of shallow features, addressing the issue of feature dilution in deep networks. Furthermore, Kwon et al. [6] optimized CNN structures for EEG emotion classification, while Moon et al. [7] proposed a CNN-based approach that explicitly incorporates brain connectivity and spatial information between electrodes. In addition to standard CNNs, methods considering the brain’s symmetrical structure [19] and 3D convolutions [20] have further enhanced spatial feature extraction.

However, standard CNNs have a fundamental limitation: they require input data to be structured as regular 2D grids. Since EEG electrodes are distributed in a non-Euclidean 3D space, mapping them onto a 2D plane often causes spatial distortion, disrupting the intrinsic topological relationships among brain regions. To address this issue, Graph Neural Networks (GNNs) have emerged as a powerful alternative capable of processing non-Euclidean data structures [21,22]. By modeling electrodes as nodes and their functional connectivity as edges, GNNs can capture global inter-channel relations without structural distortion. For example, Zhong et al. [23] and Liu et al. [24] proposed GNN-based methods that explicitly incorporate biological topology, achieving superior performance over grid-based CNN methods. Recent advancements have further refined GNN architectures. For instance, Jin et al. [25] proposed PGCN, a pyramidal GCN that aggregates features at local, mesoscopic, and global levels to better capture long-range dependencies. Similarly, Hou et al. [26] introduced DMGCN, a dual-stream, multi-level GCN that learns diverse representations across multi-metric spaces to capture hierarchical brain activities. To address the issue of subject variability, Xu et al. [27] developed DAGAM, which incorporates a domain-adversarial mechanism into a graph attention network. Furthermore, Qiu et al. [28] proposed MRGCN, which utilizes residual connections and combines short- and long-distance brain networks to extract deep emotional features. More recently, to capture continuous spatial-temporal dynamics, Hu et al. [29] proposed STRFLNet, a spatio-temporal representation fusion learning network that integrates a continuous dynamic-static graph ordinary differential equation (ODE) module with a hierarchical transformer fusion strategy.

While these spatial models excel at capturing local or global spatial patterns, they inherently overlook the temporal dynamics of brain activity. To address this, Li et al. [8] introduced Long Short-Term Memory (LSTM) networks to model sequential patterns in EEG signals [9]. Consequently, hybrid architectures integrating spatial extractors with temporal modules have been extensively explored. For instance, Shen et al. [30] proposed a 4D convolutional recurrent neural network (4D-CRNN) that integrates CNNs for spatial and spectral feature extraction with LSTMs for temporal modeling. Similarly, Henni et al. [31] proposed a hybrid framework combining an autoencoder and a CNN-LSTM network. In their approach, EEG features are reshaped into 2D matrices to apply convolutional operations, followed by LSTMs for temporal analysis. To leverage the topological advantages of graphs, Feng et al. [11] proposed a spatial-temporal graph convolutional LSTM that combines graph convolutions for spatial extraction and LSTMs for temporal modeling. Further expanding on this direction, Li et al. [12] proposed TSGCN, a temporal-spectral GCN that employs Bi-directional LSTM to extract temporal features and a dynamic GCN to capture spatial topology. Similarly, Yin et al. [32] developed a fusion model integrating graph convolutional networks with LSTMs to simultaneously capture the spatial topology and temporal dependencies of EEG signals. Likewise, Lin et al. [33] proposed CSGCN, a hybrid architecture that utilizes GCNs to model spatial connectivity and LSTMs to capture temporal evolution, demonstrating the effectiveness of spatiotemporal fusion. Supporting this direction, a recent comprehensive review by Liu et al. [34] identifies the combination of hybrid architectures and attention mechanisms as one of the most definitive technological trends in the field. They highlight that such integrated approaches are essential for effectively modeling the intricate spatiotemporal dynamics and variability inherent in EEG signals.

In addition, research has advanced toward leveraging both time-domain and frequency-domain features, reflecting the complex nature of emotions and EEG signals. In the frequency domain, specific bands such as α and β waves have been shown to correlate with emotional states. Differential Entropy (DE) has therefore been widely adopted as an effective frequency-domain feature for EEG-based emotion recognition, and its usefulness has been extensively demonstrated in prior studies [13]. Conversely, in the time domain, waveform components such as the Late Positive Potential (LPP), a sustained positive deflection starting around 400 ms after stimulus presentation, have been linked to emotional processing [35]. Schirrmeister et al. [36] demonstrated that temporal features can be learned by directly inputting raw EEG signals into a Deep ConvNet. However, approaches that rely solely on frequency-domain features lose instantaneous waveform information due to averaging over fixed windows, while time-domain-only methods preserve temporal changes but mix diverse frequency components, making it difficult to isolate emotion-related patterns. These limitations highlight the need for an integrated approach that jointly learns from both domains to fully exploit EEG characteristics for emotion recognition.

Furthermore, several studies have investigated reducing the number of electrodes to improve clinical feasibility and computational efficiency. Strategies include selecting symmetric regions or electrodes most associated with emotional processing [13]. While these methods simplify acquisition, they struggle with inter-individual variability, making it difficult to generalize across subjects. For example, Li et al. [37] explored subject-dependent models for emotion recognition, highlighting the difficulty of generalizing across individuals. To address such distribution shifts across subjects and sessions, Yu et al. [38] introduced FMLAN, a fine-grained mutual learning adaptation network that aligns features across domains by leveraging category and decision boundary information.

Finally, maximizing emotion recognition accuracy for individuals requires a mechanism to automatically search for architectures suitable for each person. Evolutionary methods such as Genetic Algorithms (GA) and Particle Swarm Optimization (PSO) have been used for subject-specific architecture search [39,40,41]. While effective, these methods require hundreds to thousands of evaluations per subject, making the computational cost extremely high and impractical. This motivates the need for differentiable architecture search methods such as DARTS, which can optimize architectures efficiently through gradient-based learning.

In terms of evaluation protocols, most existing methods rely on public EEG-based emotion datasets such as DEAP [42] and SEED [13]. The DEAP dataset provides 32-channel EEG recordings from 32 participants watching affective music video clips, whereas the SEED dataset contains 62-channel EEG recordings from 15 subjects during film-clip-based emotion induction experiments. These benchmark datasets have become de facto standards for assessing EEG-based emotion recognition models. Although the use of these benchmark datasets facilitates a certain level of comparability across methods, reported accuracies can vary substantially depending on the adopted preprocessing pipeline, feature extraction strategy, model architecture, and evaluation protocol (e.g., subject-dependent vs. subject-independent settings). As a result, strictly fair numerical comparisons between different studies remain difficult. Therefore, we summarize representative results on DEAP and SEED from recent work in Table 1 as a reference for the general performance level, rather than as an exact ranking of methods.

## 3. Foundational Techniques

This section outlines the foundational techniques that serve as the basis for the proposed method.

### 3.1. Graph Convolutional Network

Graph Convolutional Networks (GCNs) are deep learning models designed to learn from graph-structured data [15]. They generalize the convolution operation to non-Euclidean domains, where each node generates an embedding by aggregating feature information from its neighboring nodes. The fundamental aggregation process in a standard GCN is defined as follows:

(1) $$\begin{array}{cc} h_{i}^{\left(l+1\right)}=\sigma ( & \frac{1}{|d_{i}|}W^{\left(l\right)}h_{i}^{\left(l\right)}\\ \; & +\sum_{j\in N(i)}\frac{1}{\sqrt{|d_{i}\left|\right|d_{j}|}}W^{\left(l\right)}h_{j}^{\left(l\right)}). \end{array}$$

where $h_{i}^{\left(l\right)}$ denotes the feature vector of node *i* at layer *l*; N(i) represents the set of neighboring nodes of node *i*; $|d_{i}|$ is the degree of node *i*; σ is a non-linear activation function; and $W^{\left(l\right)}$ is a learnable weight matrix. By stacking multiple layers of this operation, nodes can aggregate information from a broader neighborhood range. However, standard GCNs face a limitation: The aggregation coefficients are determined solely by the static graph topology, preventing adaptive aggregation based on node features. To address this, Graph Attention Networks (GAT) introduced attention mechanisms, enabling the model to perform adaptive aggregation weighted by the specific features of neighboring nodes [43].

### 3.2. Long Short-Term Memory

Long Short-Term Memory (LSTM) is a variant of Recurrent Neural Networks (RNNs) designed for processing sequential time-series data [44]. By incorporating gating mechanisms, LSTMs overcome the long-term dependency problem inherent in traditional RNNs, enabling them to retain information across long sequences. Due to this capability, LSTMs are widely employed in emotion recognition tasks using time-series physiological data such as EEG [9,40].

### 3.3. Differentiable Architecture Search

Differentiable Architecture Search (DARTS) is an efficient Neural Architecture Search (NAS) framework that formulates architecture optimization as a differentiable problem by relaxing the discrete search space into a continuous domain [16]. Unlike conventional NAS approaches that rely on reinforcement learning or evolutionary algorithms—both of which require repeated training and evaluation of candidate architectures—DARTS significantly reduces computational cost by enabling gradient-based optimization. The core idea of DARTS is to represent each layer’s output as a weighted sum of candidate operations. Let O denote the set of candidate operations (e.g., convolution, pooling, skip connection), and $\alpha_{o}$ be the architecture parameter associated with operation o∈O. The mixed operation at layer *l* is defined as follows:

(2) $$\bar{o}\left(x\right)=\sum_{o\in O}\frac{\exp(\alpha_{o})}{\sum_{o^{\prime }\in O}\exp\left(\alpha_{o^{\prime }}\right)}o\left(x;w_{o}\right)$$

where $x\in R^{d}$ is the input feature vector (or tensor), and $w_{o}$ denotes the learnable weight vector associated with operation *o*. The softmax over α ensures differentiability and allows gradient-based optimization of architecture parameters. During the search phase, both the architecture parameters α and the network weights w are optimized jointly using gradient descent:

(3) $$\min_{w,\alpha }L_{\mathrm{train}}\left(w,\alpha \right)+L_{\mathrm{val}}\left(w,\alpha \right).$$

where $L_{\mathrm{train}}$ and $L_{\mathrm{val}}$ denote the training and validation losses, respectively. After optimization, the operation with the highest weight for each layer is selected to construct the final discrete architecture. By leveraging this approach, DARTS enables the automatic discovery of task-specific architectures with substantially lower computational overhead compared to traditional NAS methods. This capability is particularly advantageous for EEG-based emotion recognition, where inter-individual variability demands personalized architectures that would be impractical to design manually.

## 4. Proposed Method

We address the challenges of modeling electrode connectivity, capturing temporal dynamics, and reducing manual design effort by introducing three key components: a channel attention mechanism that adaptively emphasizes subject-specific important electrodes, a dual-pipeline model that integrates frequency-domain and time-domain features, and a framework for low-cost, high-speed automatic architecture optimization using Differentiable Architecture Search (DARTS).

### 4.1. Overview of the Proposed Model

**Figure 1:**
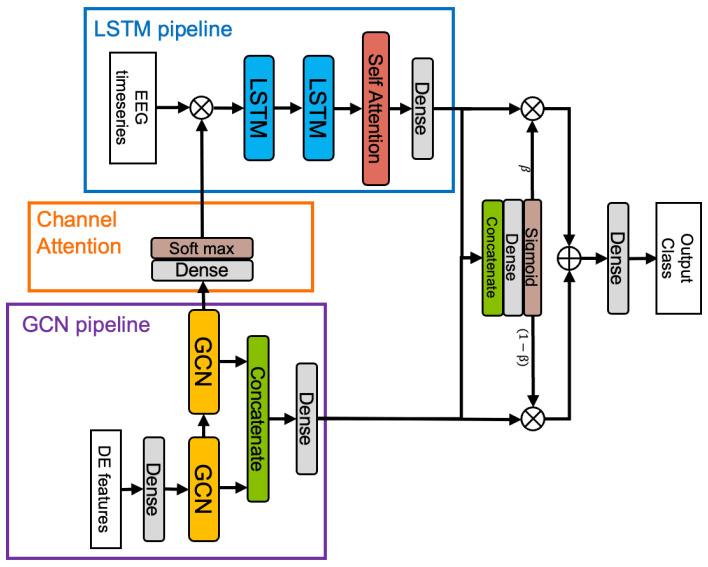
Overview of the proposed model. Blue, orange, and purple boxes indicate the LSTM-Pipeline, Channel Attention, and GCN-Pipeline, respectively. ⊗ and ⊕ denote element-wise multiplication and addition.

The proposed model is illustrated in Figure 1. It is designed to enhance emotion recognition accuracy by jointly leveraging features with distinct properties: spatial structural information and temporal dynamics. Specifically, Graph Convolutional Networks (GCNs) are applied to frequency-domain Differential Entropy (DE) features to capture topological brain structures, while Long Short-Term Memory (LSTM) networks process raw EEG signals to model temporal dependencies. By fusing these two streams, the model infers from both structural relationships and temporal fluctuations. To implement this design, we adopt a dual-pipeline architecture that processes these features in parallel. The model comprises five key components: (1) a GCN-Pipeline for extracting spatial connectivity from frequency-domain features; (2) a Channel Attention mechanism for dynamically estimating electrode importance; (3) an LSTM-Pipeline for modeling temporal dynamics from time-domain signals; (4) a Fusion module to integrate outputs from both pipelines; and (5) automatic architecture optimization using Differentiable Architecture Search (DARTS).

### 4.2. Input Feature Extraction

In this study, two complementary types of features—frequency-domain and time-domain—are extracted as inputs to the proposed model. Previous research has widely adopted Differential Entropy (DE) as a prominent frequency-domain feature due to its robustness to noise and computational efficiency [30,45,46,47]. However, DE suffers from a notable limitation: the loss of fine-grained temporal information during the extraction process, as it typically involves averaging signals over fixed time windows. To mitigate this issue, the proposed method combines DE with time-domain features derived directly from raw EEG signals. This integration enables the model to capture emotional states comprehensively from both spectral and temporal perspectives.

To construct the input representation for the proposed model, EEG signals undergo preprocessing followed by the extraction of both time-domain and frequency-domain features. First, continuous EEG data from each trial are segmented into non-overlapping windows of 3 s. Baseline correction is then applied using signals recorded immediately prior to each trial to mitigate inter-subject variability and suppress noise artifacts, following the methodology established by Yang et al. [47,48]. These baseline segments were not included in the training, validation, or testing datasets. For time-domain features, amplitude variations relative to the baseline are computed. Specifically, the median value of the baseline signal is subtracted from the raw EEG data within each segment to capture dynamic changes. The resulting signals are standardized using statistics (mean and standard deviation) computed from the training dataset only to ensure consistent scaling across inputs. For frequency-domain features, Differential Entropy (DE) is employed due to its robustness against noise and computational efficiency [30,45,46,47]. DE is calculated for each segment and its corresponding baseline across five standard frequency bands: δ (1–4 Hz), θ (4–8 Hz), α (8–14 Hz), β (14–30 Hz), and γ (30–45 Hz). Baseline correction is performed by subtracting the average DE of the baseline from that of each segment, followed by standardization.

### 4.3. GCN-Pipeline

In this model, we employ a Graph Attention Network (GAT) incorporating a Transformer-style attention mechanism within the GCN framework. Since GAT can dynamically learn edge importance directly from the data, it enables adaptive feature aggregation tailored to individual differences and emotional states. Furthermore, the GAT employed in this study utilizes a Multi-Head Attention structure. By learning independent attention weights for each head, the model captures diverse connectivity patterns among electrodes.

To incorporate structural information derived from the physical electrode configuration, we introduce a bias term $\beta_{ij}$ based on inter-electrode distances. Prior research has demonstrated the effectiveness of networks retaining approximately 20% of all possible edges [49]. Therefore, in this study, we select only the top 20% of edges based on $\beta_{ij}$ as learnable edges. The update rule for each node is defined as follows:

(4) $$\begin{array}{cc} e_{ij} & =\frac{{\left(W_{q}h_{i}^{\left(l\right)}\right)}^{⊤}\left(W_{k}h_{j}^{\left(l\right)}\right)}{\sqrt{d}}+\beta_{ij}, \end{array}$$

(5) $$\begin{array}{cc} \alpha_{ij} & =\frac{\exp(e_{ij})}{\sum_{j^{\prime }\in N\left(i\right)}\exp\left(e_{ij^{\prime }}\right)}, \end{array}$$

(6) $$\begin{array}{cc} h_{i}^{\left(l+1\right)} & =\sigma \;\left(\sum_{j\in N(i)}\alpha_{ij}\;W_{v}h_{j}^{\left(l\right)}\right). \end{array}$$

where $h_{i}^{\left(l\right)}$ denotes the feature vector of node *i* at layer *l*; *d* represents the dimensionality; and N(i) is the set of neighboring nodes connected to node *i* via learnable edges. The term $\beta_{ij}$ is a bias added to the attention score, calculated based on the physical distance between electrodes, and $\alpha_{ij}$ represents the edge importance from node *i* to node *j*.

Following prior work, the bias term $\beta_{ij}$ is defined using a Gaussian kernel based on the physical distance between electrodes as follows [50]:

(7) $$\begin{array}{c} \beta_{ij}=\exp\;\left(-\frac{d_{ij}^{2}}{\tau^{2}}\right). \end{array}$$

where $p_{i}\in R^{3}$ denotes the 3D coordinates of electrode *i*; $d_{ij}={∥p_{i}-p_{j}∥}_{2}$ represents the Euclidean distance; and τ is the scaling factor of the Gaussian kernel.

### 4.4. Channel Attention Mechanism

The Channel Attention (CA) mechanism is introduced to automatically identify subject-specific salient electrodes and emphasize their contribution. By incorporating the computed electrode importance into the input features of the LSTM-Pipeline, the model can adaptively focus on the most informative brain regions for each individual, thereby addressing inter-subject variability and improving model robustness. While the GAT module learns the importance of the *connections* (edges) between electrodes, the CA mechanism explicitly learns the importance of the *electrodes themselves* (nodes). In this mechanism, an importance score $s_{i}$ for electrode *i* is computed from the output of the second GAT layer, $h_{i}^{\left(2\right)}$. These scores are then normalized using the Softmax function to obtain attention weights ${\mathrm{att}}_{i}$, as defined by the following equation:

(8) $${\mathrm{att}}_{i}=\mathrm{Softmax}\left(s_{i}\right),\;s_{i}=w^{⊤}h_{i}^{\left(2\right)}.$$

Here, $w\in R^{d}$ is a learnable weight vector used to project the node feature $h_{i}^{\left(2\right)}$ into a scalar score $s_{i}$. This parameter determines how strongly each feature dimension contributes to the attention score. Since the sum of Softmax outputs across channels equals 1, the signals may be excessively attenuated. To prevent this, the attention weights are scaled in the implementation such that their mean value becomes 1.

These attention weights are then used to gate the time-domain features that serve as input to the LSTM-Pipeline. The gating operation is formulated as follows:

(9) $$x_{\mathrm{gated}}\left(t,i\right)=x_{\mathrm{raw}}\left(t,i\right)\cdot \left[\left(1-\beta \right)+\beta \cdot {\mathrm{att}}_{i}\right].$$

where *t* represents the time step, and β∈(0,1) is a learnable mixing coefficient that controls the extent to which the attention weights are applied. Here, $x_{\mathrm{raw}}\left(t,i\right)$ denotes the original EEG amplitude for electrode *i* at time step *t*, and $x_{\mathrm{gated}}\left(t,i\right)$ represents the attention-modulated signal after applying the gating mechanism. Through this mechanism, information about salient electrodes learned by the GCN-Pipeline is effectively reflected in time-domain processing.

### 4.5. LSTM-Pipeline

The LSTM-Pipeline is designed to learn time-domain features of EEG signals effectively. In constructing this pipeline, we adopted the approach proposed by Oka et al. [40]. They proposed an emotion estimation model that incorporates a temporal attention mechanism into an LSTM, dynamically emphasizing critical time steps relevant to emotional states. Their experiments demonstrated the effectiveness of this approach. In this study, we determined that this LSTM architecture with temporal attention is highly effective for learning time-domain features and, therefore, employed it as the foundation of our LSTM-Pipeline.

### 4.6. Fusion Mechanism

The Fusion mechanism is designed to integrate features extracted by the GCN-Pipeline and the LSTM-Pipeline, thereby enabling the effective utilization of both structural and temporal information. Specifically, a learnable gating mechanism is employed to dynamically weight and merge the outputs of each pipeline. Let $z_{\mathrm{GCN}}$ be the output of the GCN-Pipeline and $z_{\mathrm{LSTM}}$ be the output of the LSTM-Pipeline. The fused output $z_{\mathrm{fused}}$ is calculated as follows:

(10) $$\begin{array}{cc} g & =\sigma (w^{⊤}\left[z_{\mathrm{GCN}};z_{\mathrm{LSTM}}\right]+b), \end{array}$$

(11) $$\begin{array}{cc} z_{\mathrm{fused}} & =\left(1-g\right)z_{\mathrm{LSTM}}+gz_{\mathrm{GCN}}. \end{array}$$

where σ denotes the sigmoid function, and w and *b* are learnable parameters. The gate *g* is computed dynamically for each sample, adaptively adjusting the contribution of each pipeline’s output.

### 4.7. Hyperparameter Optimization via DARTS

To maximize model performance and adapt to inter-individual variability, we employ the DARTS framework described in Section 3.3 to optimize key architectural hyperparameters. Unlike manual tuning, which is computationally expensive and prone to suboptimal configurations, DARTS enables automatic discovery of architectures tailored to subject-specific characteristics through gradient-based optimization.

The search space was carefully designed based on preliminary experiments to strike a balance between search flexibility and computational feasibility. Specifically, the candidate value ranges were set to sufficiently cover the parameter spaces typically used in existing EEG emotion recognition studies, extending from minimal to sufficiently large capacities. For the LSTM pipeline, the number of recurrent units and dense layer units is varied to capture temporal complexity across different scales. In the GCN pipeline, the search space includes the number of graph convolutional units and the number of attention heads, allowing the model to adjust its capacity to learn spatial connectivity patterns. The complete search space is summarized in Table 2. Furthermore, because the output dimensions of the two pipelines are optimized independently, a Dense layer is introduced prior to the fusion mechanism. This layer projects the GCN pipeline’s output to match the dimensionality of the LSTM pipeline, ensuring effective integration of structural and temporal features without introducing bias toward either modality.

## 5. Performance Evaluation

In this section, we conduct experiments to verify the effectiveness of the proposed model. First, Section 5.1 describes the experimental settings common to all experiments in this section. Subsequently, Section 5.2 and onwards present the details and results of each specific verification experiment.

### 5.1. Experimental Settings

#### 5.1.1. DEAP Dataset

**Figure 2:**
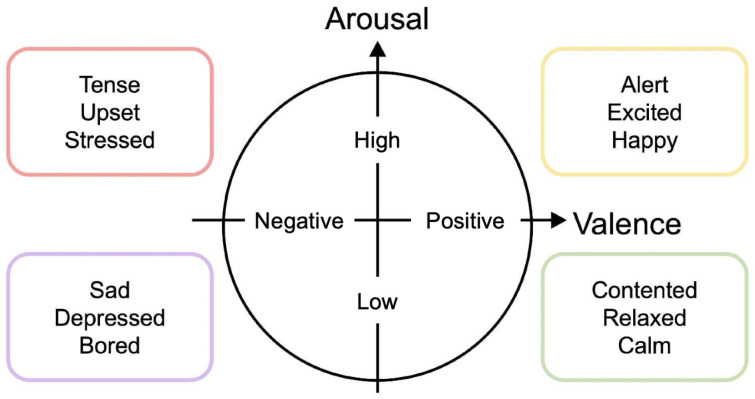
Russell’s circumplex model.

In this study, we utilize the DEAP dataset [42], a widely used benchmark for emotion recognition. The dataset comprises physiological signals recorded from 32 participants while they viewed 40 one-minute music videos. EEG signals were collected from 32 electrodes positioned according to the international 10–20 system. Each trial includes a 3-s baseline recorded prior to video presentation and a 60-s EEG segment captured during viewing. After each video, participants rated their emotional state on a 9-point Likert scale for four dimensions, including Valence and Arousal, based on Russell’s circumplex model (Figure 2). In this work, we focus on Valence, which represents the degree of pleasantness, and Arousal, which represents emotional intensity. We employed a window-level splitting strategy consistent with standard methodologies in EEG emotion recognition research [19,24,33,40]. Specifically, continuous EEG signals were segmented into non-overlapping windows. These segments were then randomly shuffled and split into training, validation, and testing sets at a 6:2:2 ratio, a configuration designed to rigorously evaluate model performance.

#### 5.1.2. Task Definition and Evaluation Metrics

The DEAP dataset provides self-assessment ratings ranging from 1 to 9 for the Valence and Arousal axes. In this study, we treat emotion recognition as a four-class classification task. Specifically, using a threshold of 5 for both axes, Valence and Arousal are each classified into two classes: High (≥5) and Low (<5). By combining these classes, the emotional states are categorized into four distinct classes: High Valence–High Arousal (HVHA), Low Valence–High Arousal (LVHA), Low Valence–Low Arousal (LVLA), and High Valence–Low Arousal (HVLA). We adopted a subject-dependent approach, training individual models for each participant. Accuracy was employed as the performance metric. The final evaluation metric was determined as the average accuracy calculated across all 32 subjects.

#### 5.1.3. Parameter Settings

The model was trained using the frequency-domain and time-domain features defined in Section 4.2. We employed the Adam optimizer with a learning rate of 0.001, a batch size of 32, and the number of training epochs set to 150. All experiments were conducted on a single NVIDIA A100 GPU. The architecture search via DARTS was executed for 40 epochs. Thanks to gradient-based optimization and a high-performance computing environment, the search process completes in approximately 170 s per trial, which is significantly more efficient than evolutionary computation methods that typically require hours. To ensure the robustness of the search results, we executed this process five times independently with different random seeds. During this search phase, the training and validation datasets were temporarily combined and equally divided into two subsets: one for updating the network weights and the other for updating the architecture parameters. After determining the optimal architecture from the search, we retrained the model. For this retraining phase, we reverted to the standard data split, utilizing 60% for training and 20% for validation, to identify the architecture that achieved the highest validation accuracy.

### 5.2. Experimental Results

In this study, we addressed multiple challenges in EEG-based emotion recognition by proposing a model composed of five key components, as described in Section 4. To evaluate the individual contribution and effectiveness of each component, we conducted an ablation study. To validate the proposed model, we defined several variants by incrementally adding components and compared their performance. The detailed configuration of each variant is presented in Table 3. It is important to note that the Fusion mechanism was applied only to configurations that incorporated both the LSTM pipeline and the GCN pipeline. For configurations using a single pipeline, the output of that pipeline was fed directly into the classifier, bypassing the Fusion mechanism. Consequently, Models 1 and 2 did not employ Fusion, whereas Models 3 through 5 did. For Models 1 through 4, which do not include DARTS, the fixed hyperparameters were configured as follows: In the LSTM-Pipeline, the first and second LSTM layers were set to 32 and 64 units, respectively, with an output Dense layer of 32 units. In the GCN-Pipeline, the first and second GAT layers were set to 32 and 64 units, respectively; both layers utilized two attention heads, and the output Dense layer consisted of 32 units. The Fusion mechanism was employed in models that combine the two pipelines to integrate their respective features.

Table 3 presents the results of the ablation study. Consistent with the task definition in Section 5.1.2, these results are based on the four-class classification task (HVHA, HVLA, LVHA, LVLA) derived from the Valence and Arousal dimensions of the DEAP dataset. First, Model 3 achieved significantly higher accuracy compared to both Model 1 and Model 2. If the information captured by the two pipelines were redundant, such a substantial performance improvement would not be expected. Therefore, this result strongly indicates that the structural information captured by the GCN and the temporal information captured by the LSTM function complementarily. This demonstrates that the proposed dual-pipeline architecture effectively learns EEG characteristics by modeling spatial connectivity in the frequency domain and temporal dynamics in the time domain.

Next, Model 4, which incorporates the Channel Attention mechanism, showed a slight improvement in accuracy over Model 3. Although the margin of improvement was limited—likely because the Fusion mechanism in Model 3 was already highly effective—the introduction of the Channel Attention mechanism provides a significant advantage in terms of interpretability. By visualizing and interpreting subject-specific important electrodes, the mechanism enhances the practical utility of deep learning models, which often suffer from being black boxes. The validity of the importance scores derived from the Channel Attention mechanism and their practical utility are verified in Section 6.1 and Section 6.2.

Finally, we compare Model 5, optimized via DARTS, with Model 4, which used fixed hyperparameters. Model 5 achieved the highest performance in terms of both mean and maximum accuracy. This indicates that DARTS successfully explored hyperparameters tailored to the data characteristics of individual subjects, unlocking potential performance that fixed parameters could not reach.

Regarding the comparison with state-of-the-art methods presented in Table 1, our model achieves competitive performance (0.9317) compared to recent baselines such as CSGNN [33] (0.9100). Although PSO-LSTM [40] reports slightly higher accuracy (0.9404), it relies on Particle Swarm Optimization, an evolutionary algorithm that typically requires extensive computational time and many iterations to converge. In contrast, our DARTS-based approach utilizes gradient-based optimization, allowing the architecture search to be completed in approximately 170 s on a standard GPU. We argue that this trade-off—achieving competitive accuracy while significantly improving search efficiency—offers a substantial practical advantage, particularly for real-world applications that require frequent retraining or rapid personalization.

**Figure 3:**
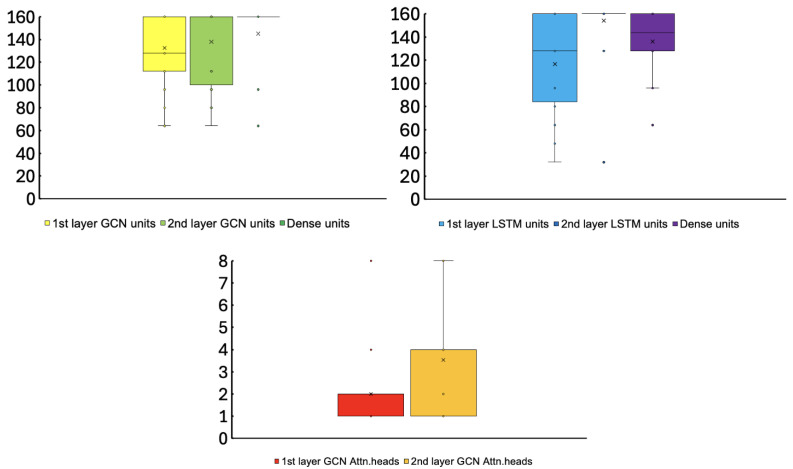
Box-and-whisker diagram for hyperparameters of all 32 subjects.

**Figure 4:**
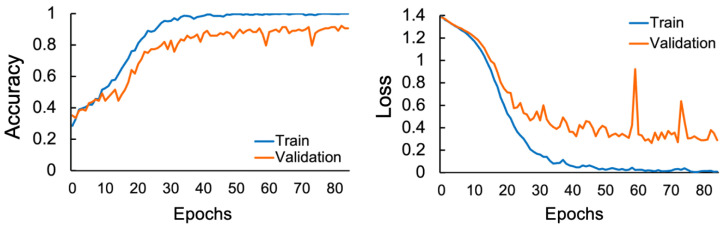
Learning curve of subject 17.

Figure 3 shows box-and-whisker plots of optimized hyperparameters across 32 subjects. Most subjects (31/32) converged to unique architectures, confirming subject-specific optimization. However, the lower minimum accuracy and higher standard deviation observed in Table 3 indicate that the optimization was insufficient for a subset of subjects. Figure 4 illustrates Loss and Accuracy during retraining for Subject 17, revealing overfitting: training metrics improve continuously, while validation metrics plateau around Epochs 20–30, creating a large gap.

Similar optimization difficulties for this subject were reported by Oka et al. [40], who used PSO and attributed them to local optima, suggesting enhanced search strategies. The fact that both PSO and DARTS struggled implies that non-stationarity or distribution shifts in this subject’s EEG data are more critical than the choice of optimization method. This ablation study confirms the contribution of each component in the proposed model.

### 5.3. Cross-Dataset Validation on SEED

Table 4 presents the comparison results. The model achieved a mean accuracy of 0.9034 on the SEED dataset. While this performance is lower than PSO-LSTM (0.9732) [40], which employs computationally intensive evolutionary optimization, it remains competitive with many recent approaches while maintaining the efficiency of gradient-based architecture search.

Notably, the accuracy on SEED (0.9034) is lower than that achieved on DEAP (0.9317), despite SEED having nearly double the number of electrodes (62 vs. 32). We attribute this discrepancy to the over-smoothing problem inherent in GCNs applied to dense montages.

While DEAP’s 32-node graph maintains sufficient feature diversity through moderate connectivity, SEED’s 62-node graph—even after thresholding—results in a higher node degree, causing node representations to converge to indistinguishable values after multiple message-passing steps. This excessive mixing reduces the discriminative power of the learned features. Additionally, the larger number of electrodes may introduce redundant or noisy channels unrelated to emotional processing, hindering the Channel Attention mechanism’s ability to effectively isolate critical brain regions.

These findings indicate that for high-density EEG datasets like SEED, sparse graph construction strategies, hierarchical graph pooling, or additional regularization may be necessary to fully leverage spatial information while mitigating over-smoothing effects.

## 6. Discussion

### 6.1. Channel Attention

This section aims to validate the plausibility of importance scores derived from the Channel Attention mechanism and assess the utility of electrode selection based on these scores. Although the accuracy gain from introducing Channel Attention was modest, the mechanism provides practical value by identifying subject-specific salient electrodes, enabling adaptive electrode reduction. In both experiments, the number of retained electrodes was set as K∈{1,2,…,32}. The model was trained using all 32 electrodes, and electrode importance was computed from the validation data. Specifically, we compared performance when reducing the test data to *K* electrodes using three methods: retaining the top *K* most important electrodes (Top-*K*), the bottom *K* least important electrodes (Bottom-*K*), and *K* randomly selected electrodes (Random-*K*).

**Figure 5:**
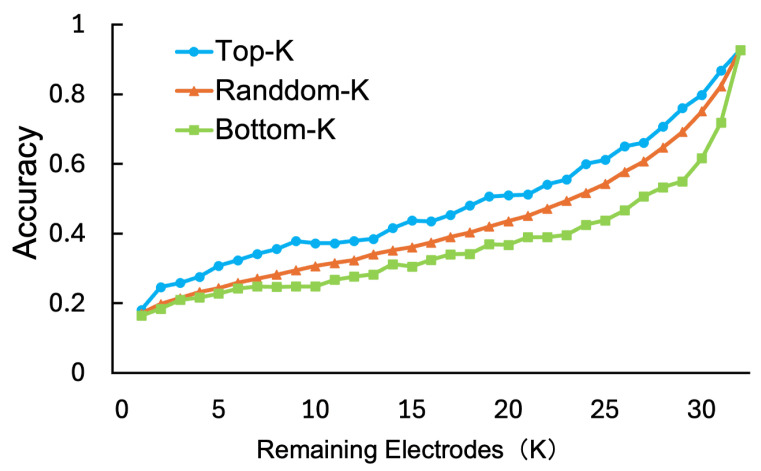
Electrode importance validation.

Figure 5 shows the relationship between the number of retained electrodes *K* and Accuracy. As shown in the figure, with the exception of K=4, the performance followed the order of Top-*K* > Random-*K* > Bottom-*K*. This result implies that the important electrodes identified by the Channel Attention mechanism are indispensable for maintaining accuracy. In particular, at K=31, the accuracy of the Bottom-*K* condition dropped sharply, whereas the accuracy decrease in the Top-*K* condition was gradual. This indicates that removing the most important electrode causes a significant drop in performance, while removing the least important electrode has a minimal impact. Specifically, the sharp performance drop at K=31 in the Bottom-*K* condition confirms that the top-ranked electrode plays a decisive role. Importance scores were averaged across validation samples to mitigate transient artifacts, such as eye blinks. Furthermore, when analyzing the top-1 electrode for each of the 32 subjects, we observed that the most frequently selected electrodes spanned not only frontal regions (e.g., AF4, F7) associated with critical frequency bands and channels for emotion processing [13], but also parietal areas (e.g., P4). Notably, we also observed high importance scores in occipital electrodes in specific cases, reflecting inter-individual variability in neural responses. This spatial diversity suggests the model captures meaningful neural patterns rather than localized artifacts.

**Figure 6:**
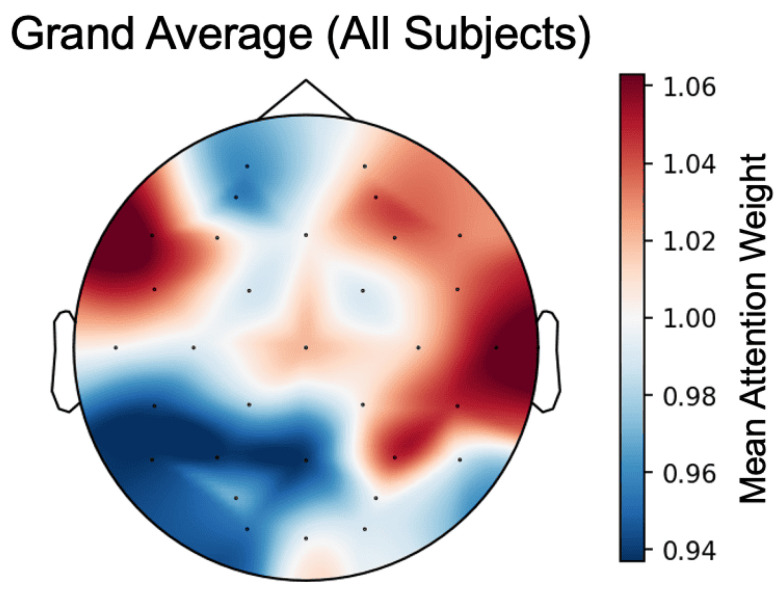
Average of attention maps. Dots indicate electrode positions.

Figure 6 presents the spatial distribution of important electrodes averaged across all subjects. Here, importance scores were averaged across subjects to identify regions with consistently high quantitative scores. The channels with high importance across all subjects were T8, F7, CP6, P4, and FC5. In neuroscience, the frontal lobes (e.g., F7) are known to be involved in the regulation and judgment of emotional valence, while the temporal lobes (e.g., T8) play a crucial role in processing emotional facial information and social cognition [13,51,52]. Furthermore, the right parietal-temporal regions (CP6, P4) have been reported to be strongly associated with the processing of emotional arousal [53]. The fact that the proposed model autonomously identified these emotion-related regions as “important” suggests that the model is learning physiologically meaningful brain activity patterns rather than noise.

**Figure 7:**
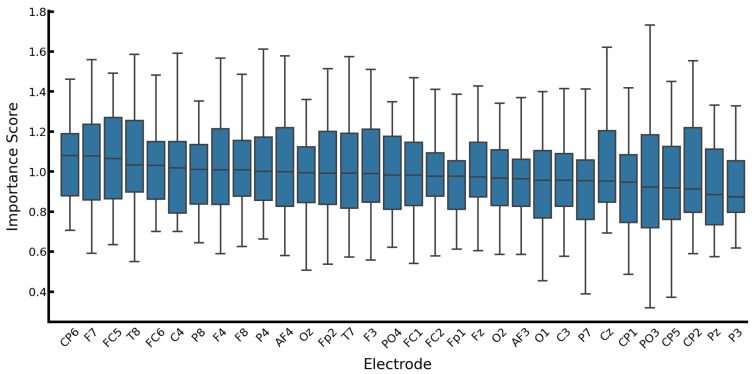
Distribution of electrode importance scores across all 32 subjects.

Additionally, Figure 7 illustrates the distribution of importance scores across all 32 subjects to assess potential biases. The substantial variance (wide whiskers) indicates that the model does not rely on a fixed subset of electrodes but adaptively identifies informative regions specific to each individual’s neural patterns.

Moreover, we investigated individual differences in attention distribution. We selected Subject 1 and Subject 19, who exhibited the lowest correlation in attention distribution (r=−0.87). Note that the emotion recognition accuracy using all electrodes was extremely high for both subjects (Subject 1: 90.37%, Subject 19: 98.34%), indicating that the model successfully learned emotional features for both.

**Figure 8:**
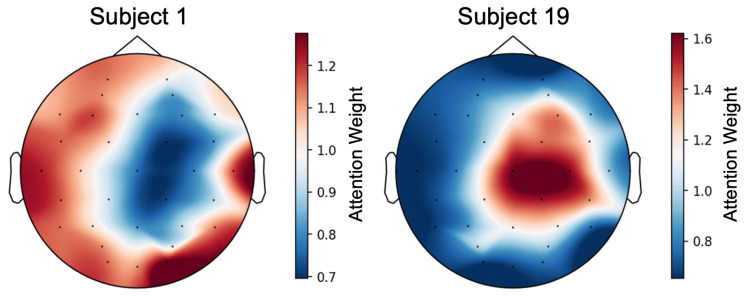
Attention maps of Subject 1 and 19. Dots indicate electrode positions.

Figure 8 shows the topographical maps for both subjects. Despite achieving high accuracy in both cases, the focused regions are contrasting. Subject 1 places importance on the visual cortex (O2) and temporal regions (T7, T8), suggesting a strong reliance on visual and auditory information processing in response to emotion-eliciting stimuli. In contrast, Subject 19 prioritizes regions near the sensorimotor cortex (Cz, C4) and the central parietal area, suggesting that interoceptive sensations accompanying emotional arousal may be used as cues rather than visual input itself.

Thus, even when high-precision estimation is possible, the brain regions used as primary cues can differ significantly between subjects. This fact highlights the risk that fixed electrode selection may discard critical information for specific subjects, strongly supporting the necessity of subject-adaptive electrode selection, as demonstrated in Section 6.2. Therefore, the validity of the electrode importance scores was demonstrated, confirming the utility of the Channel Attention mechanism in deriving these scores.

### 6.2. Adaptive Electrode Selection

**Figure 9:**
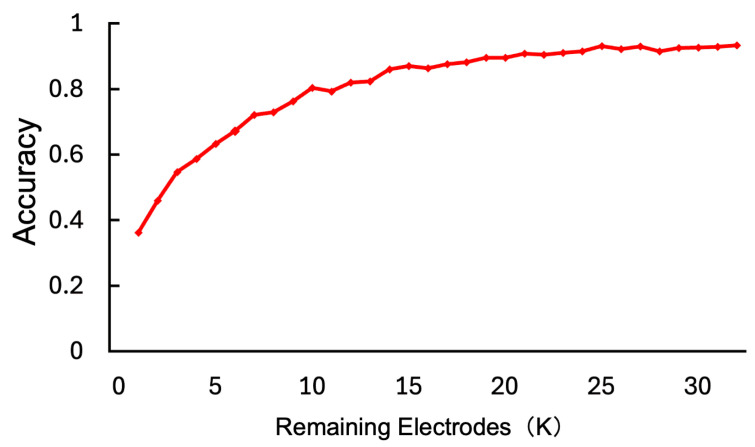
Adaptive electrode reduction. The red line indicates the accuracy of the proposed method as a function of the number of retained electrodes.

In this section, we evaluate the impact of adaptive electrode reduction based on importance scores derived from the Channel Attention mechanism. Specifically, we retrained the proposed model using only the top *K* important electrodes and tested it on data containing the same *K* electrodes. To ensure fairness, the architecture was fixed to the one optimized for 32 electrodes. The results are shown in Figure 9 and Table 5. The horizontal axis represents the number of retained electrodes *K*, and the vertical axis represents accuracy. As illustrated, high-precision emotion recognition is achievable even with a small number of electrodes through importance-based selection. Notably, at *K* = 10 (31% of total), accuracy reached 80.35%, and at *K* = 25 (78% of total), accuracy reached 93.08%. This demonstrates that over 80% accuracy can be achieved with only about 30% of the electrodes, and performance nearly equivalent to using all electrodes (93.17%) can be realized with about 80%. These results confirm the utility of adaptive electrode selection tailored to each subject.

## 7. Conclusions and Future Work

We introduced a dual-pipeline architecture that integrates frequency-domain and time-domain EEG features through GCN and LSTM, complemented by a Channel Attention mechanism for subject-specific adaptation. Additionally, we employed Differentiable Architecture Search (DARTS) to automatically discover optimal architectures tailored to individual EEG characteristics. Experimental results demonstrated that the proposed framework achieves competitive accuracy compared to state-of-the-art methods, underscoring its potential for robust and personalized emotion recognition. While PSO-LSTM can achieve higher accuracy, its days-long evolutionary search limits practicality; our minutes-level DARTS search attains competitive accuracy with a fraction of the cost, making it the preferable choice for time- and compute-constrained deployment.

Future research should focus on addressing two critical challenges revealed by our findings. First, although DARTS improved mean accuracy, it also reduced the minimum accuracy and increased the standard deviation across subjects. This variability suggests that the search process may occasionally select unstable architectures for individuals with complex EEG patterns. Developing more robust optimization strategies, such as incorporating regularization or uncertainty-aware mechanisms, will be essential to ensure consistent performance across diverse populations.

Second, robustness under extremely low-electrode conditions remains a significant limitation. Our experiments showed that performance deteriorates sharply when fewer than thirteen electrodes are available, in contrast to the relatively stable results reported by Lin et al. [33]. While Lin et al.’s simpler model structure exhibits resilience under limited information, our expressive architecture requires a minimum threshold of input richness to fully realize its potential. To overcome this, future work could integrate constraints such as computational cost, parameter count, or connectivity sparsity into the DARTS search process. Such constraints would not only enable the discovery of lightweight architectures for low-electrode scenarios but also mitigate over-smoothing effects in high-density settings like SEED. Such improvements are crucial for practical applications where portability and user comfort are paramount. Additionally, investigating the model’s performance in subject-independent (cross-subject) settings is another essential direction. While the present study adopted a subject-dependent approach to maximize personalized accuracy, cross-subject validation would provide valuable insights into the generalizability of learned features and the model’s ability to capture emotion-related patterns that transfer across individuals. Beyond these challenges, expanding the proposed framework to multimodal emotion recognition represents a promising direction. Combining EEG with complementary physiological signals such as ECG, EDA, or EMG could enhance robustness and ecological validity, particularly in real-world environments. Furthermore, personalization remains an open problem: incorporating meta-learning or continual learning strategies could allow the system to adapt dynamically as more subject-specific data becomes available, facilitating deployment in clinical and consumer-facing contexts. 

## Figures and Tables

**Table 1:** Related Work.

Research	Model	DEAP	SEED
Deng et al. [19]	SFE-Net	0.8634	0.9919
Liu et al. [24]	MST-GNN	0.9231	-
Hu et al. [29]	STRFLNet	-	0.9642
Marjit et al. [39]	MLP	0.8352	-
Yu et al. [38]	FMLAN	-	0.9194
Oka et al. [40]	PSO-LSTM	0.9404	0.9732
Lin et al. [33]	CSGNN	0.9100	0.9022

**Table 2:** Search space.

Pipeline	Parameter	Search Space
LSTM	LSTM Units	{32, 48, 64, 80, 96, 128, 160}
	Dense Units	{64, 96, 128, 160}
GCN	GCN Units	{32, 48, 64, 80, 96, 112, 128, 160}
	Attn. Heads	{1, 2, 4, 8}
	Dense Units	{64, 96, 128, 160}

**Table 3:** Results of the Ablation Study.

Model	Components				Accuracy			
	LSTM-P	GCN-P	CA	DARTS	Mean	Std	Max	Min
1	✓				0.8490	0.0858	0.9812	0.6625
2		✓			0.4307	0.1067	0.6813	0.2000
3	✓	✓			0.9199	0.0444	0.9813	0.8125
4	✓	✓	✓		0.9277	0.0427	0.9875	0.8000
5	✓	✓	✓	✓	**0.9317**	0.0601	**1.0000**	0.7773

LSTM-P: LSTM-Pipeline, GCN-P: GCN-Pipeline, CA: Channel Attention Mechanism.

**Table 4:** Performance comparison of the proposed method on DEAP and SEED datasets.

Dataset	Task	Accuracy			
		Mean	Std	Max	Min
DEAP	4-class	0.9317	0.0601	1.0000	0.7773
SEED	3-class	0.9034	0.0460	0.9555	0.7874

**Table 5:** Accuracy with importance-based electrode reduction.

# Electrodes	Proposed Method
32	0.9317
26	0.9212
19	0.8955
13	0.8232
6	0.6718

## Data Availability

The DEAP dataset can be found at https://www.eecs.qmul.ac.uk/mmv/datasets/deap/ (accessed on 18 July 2023).
